# Clinical and Radiological Outcomes of Oral Bisphosphonate Therapy Combined with Conservative Treatment in Subchondral Bone Marrow Edema of the Knee: A Retrospective Cohort Study

**DOI:** 10.3390/jcm15176529

**Published:** 2026-08-24

**Authors:** Goker Yurdakul, Murat Korkmaz, Irfan Karadak, Abdullah Iyigun, Muhammed Kazez, Nezih Yayli, Mustafa Fatih Erkoc, Fatih Golgelioglu

**Affiliations:** 1 Department of Orthopedics and Traumatology, Yozgat Bozok University, 66100 Yozgat, Türkiye; goker.yurdakul@bozok.edu.tr (G.Y.); murat.korkmaz@bozok.edu.tr (M.K.); irfankaradak@gmail.com (I.K.); 2 Department of Orthopedics and Traumatology, Umraniye Training and Research Hospital, 34764 Istanbul, Türkiye; abdullah.iyigun@saglik.gov.tr; 3 Department of Orthopedics and Traumatology, Elazig Fethi Sekin City Hospital, 23000 Elazig, Türkiye; mkzz23@hotmail.com; 4 Department of Radiology, Yozgat Bozok University, 66100 Yozgat, Türkiye; nezih.yayli@bozok.edu.tr (N.Y.); mustafafatih.erkoc@bozok.edu.tr (M.F.E.)

**Keywords:** alendronate, bisphosphonate, bone marrow edema, conservative treatment, knee, good health and well-being

## Abstract

**Background/Objectives:** Subchondral bone marrow edema (BME) of the knee often causes persistent pain despite conservative treatment, and evidence supporting oral bisphosphonates is limited. We compared conservative treatment alone with conservative treatment plus oral alendronate. **Methods**: This retrospective cohort study included 82 patients with MRI-confirmed subchondral BME of the knee treated between January 2020 and January 2025. Patients received conservative treatment alone (*n* = 44) or conservative treatment plus alendronate 70 mg weekly for three months (*n* = 38). Visual Analog Scale (VAS) and Western Ontario and McMaster Universities Osteoarthritis Index (WOMAC) scores were assessed at baseline and three months. Radiological response was assessed on follow-up MRI as the percentage reduction in BME area. **Results**: Both groups improved significantly at three months. The alendronate group showed greater reductions in VAS (4.0 ± 1.4 vs. 3.2 ± 1.3 points, *p* = 0.009) and total WOMAC scores (25.5 ± 8.9 vs. 19.8 ± 8.6 points, *p* = 0.002). Median time to full weight-bearing was shorter with alendronate (7 vs. 9 weeks, *p* = 0.021), and complete BME regression was more frequent (42.1% vs. 18.2%, Fisher’s exact *p* = 0.028). After adjustment for Kellgren–Lawrence grade, alendronate was associated with a favorable radiological response (OR = 3.66, 95% CI: 1.18–11.31, *p* = 0.024). No serious adverse events were recorded. **Conclusions**: Adding oral alendronate to conservative treatment was associated with greater clinical improvement, earlier full weight-bearing, and a more favorable radiological response at three months. Prospective randomized studies are needed to confirm these findings.

## 1. Introduction

Subchondral bone marrow edema (BME) of the knee is a painful condition characterized by the accumulation of interstitial fluid within the subchondral bone, typically identified as hyperintense signal changes on fat-suppressed magnetic resonance imaging (MRI) sequences [1,2]. It may arise in the context of various underlying conditions, including osteoarthritis, subchondral insufficiency fractures, and transient osteoporosis, and often results in significant weight-bearing pain and functional limitations disproportionate to plain radiographic findings [3,4]. Subchondral bone marrow lesions are common across the spectrum of knee disease: in a population-based cohort of adults with knee pain, they were present in 38% of patients with pre-radiographic osteoarthritis and in 71% of those with radiographic osteoarthritis [5], and were detectable in approximately half of adults older than 50 years even in the absence of radiographic osteoarthritis [6]. Recognized risk factors include increasing age [6], mechanical limb malalignment, and vitamin D deficiency, and the presence of such lesions is a potent risk factor for subsequent structural progression of knee osteoarthritis [7,8]. The condition imposes a considerable burden on patients, frequently interfering with daily activities and quality of life, yet its optimal management remains poorly defined.

The initial approach to treatment is conservative management, encompassing activity modification, partial weight-bearing, nonsteroidal anti-inflammatory drugs (NSAIDs), and physiotherapy. Despite these measures, symptoms may persist for several months, and a subset of patients fails to achieve satisfactory clinical or radiological improvement [1,4]. This underscores the need for adjunctive therapeutic strategies capable of accelerating edema resolution and alleviating pain more effectively.

Bisphosphonates have emerged as a potential treatment option for subchondral BME owing to their ability to inhibit osteoclast-mediated bone resorption and modulate subchondral bone turnover [9]. However, the available evidence is not uniform across administration routes or individual agents. To date, the most robust data pertain to intravenous formulations: zoledronic acid demonstrated efficacy for nonmalignant painful bone marrow lesions in a triple-blind, randomized, placebo-controlled trial [10], and intravenous ibandronate and neridronate have been associated with meaningful pain relief and radiological improvement in patients with bone marrow edema [9,11,12]. Evidence addressing the knee joint specifically is also accumulating: intravenous ibandronate provided effective pain control in patients with bone marrow edema of the knee [11], bisphosphonate therapy achieved complete regression of edema in 40% of patients with bone marrow edema syndrome of the knee and foot at three months [12], and a systematic review of bone marrow lesions of the knee reported complete resolution in approximately half of bisphosphonate-treated patients within a mean of three months [13]. In contrast, the evidence supporting oral bisphosphonates is considerably more limited, consisting mainly of small and heterogeneous series that differed with respect to agents, dosing regimens, and anatomical sites [14,15]. To our knowledge, no controlled study has specifically evaluated weekly oral alendronate as an adjunct to conservative treatment in subchondral BME of the knee, although alendronate offers practical advantages over intravenous agents in terms of accessibility, outpatient applicability, and cost. Whether these practical advantages are accompanied by clinical and radiological benefits comparable to those reported for intravenous formulations therefore remains unresolved.

To address this gap, the present study compared the clinical and radiological outcomes of conservative treatment alone versus conservative treatment combined with weekly oral alendronate in patients with subchondral BME of the knee. We hypothesized that the addition of oral bisphosphonate therapy to standard conservative treatment would result in superior clinical and radiological outcomes compared to conservative treatment alone. The primary outcome was the change in Visual Analog Scale (VAS) pain score from baseline to the three-month follow-up; secondary outcomes included changes in Western Ontario and McMaster Universities Osteoarthritis Index (WOMAC) scores, time to return to full weight-bearing, and the radiological response on follow-up MRI.

## 2. Materials and Methods

### 2.1. Study Design

This was a single-center, retrospective cohort study conducted at a university hospital to compare the clinical and radiological outcomes of conservative treatment with or without oral bisphosphonates in patients with subchondral bone marrow edema of the knee. The study protocol was approved by The Institutional Ethics Committee of Yozgat Bozok University (Approval Date: 2 July 2025; Approval Number: 2025-GOKAEK-2513_2025.07.02_566), and informed consent to participate was obtained from all of the participants in the study. The study was reported in accordance with the Strengthening the Reporting of Observational Studies in Epidemiology (STROBE) guidelines for cohort studies (Table S1).

### 2.2. Patient Selection

Patients diagnosed with subchondral bone marrow edema of the knee between January 2020 and January 2025 were retrospectively identified from the institutional electronic medical record system. The study was initiated after institutional ethics committee approval had been obtained (2 July 2025); the electronic medical record database was queried, and all clinical and radiological data were extracted and analyzed, after this approval date. All data had been documented during routine clinical care and were collected retrospectively for the purposes of the present study. The diagnosis was confirmed by MRI, demonstrating characteristic subchondral signal alterations, including low signal intensity on T1-weighted images and high signal intensity on fat-suppressed T2-weighted sequences.

Patients were eligible for inclusion if they were between 18 and 70 years of age, had MRI-confirmed subchondral bone marrow edema of the knee, received either conservative treatment alone or conservative treatment combined with oral bisphosphonate therapy, and had documented concordance between the anatomical location of BME on MRI and the site of predominant pain on physical examination, as recorded in the clinical notes. Clinical and radiological assessments performed at the three-month follow-up visit were included in the final analysis. Only patients with complete clinical, radiological, and treatment records were included.

Patients were excluded if they had acute fractures around the knee, inflammatory arthritis, septic arthritis, osteomyelitis, malignancy, osteonecrosis, and prior osteotomy around the affected knee. Patients receiving systemic corticosteroid therapy or with any previous use of antiresorptive or other bone-acting agents (e.g., bisphosphonates, denosumab, teriparatide) were also excluded, to avoid confounding of subchondral bone metabolism. Patients with traumatic bone marrow edema secondary to ligamentous or meniscal injury, as well as those with diffuse bone marrow edema not confined to the subchondral region, were also excluded. In addition, patients with advanced osteoarthritis (Kellgren–Lawrence grade 4) or subchondral insufficiency fractures associated with advanced articular surface collapse were excluded. Patients were also excluded if physical examination findings documented in the clinical records revealed signs attributable to concomitant structural pathology, including positive meniscal provocation tests or ligamentous instability. The presence of mild-to-moderate degenerative meniscal signal changes on MRI (grade 1–2) without corresponding clinical findings was not considered an exclusion criterion; however, grade 3 meniscal tears with concordant clinical findings were excluded. Because bone marrow edema is an MRI finding rather than a single disease entity, the study population was defined as patients with non-traumatic subchondral BME of the knee and thus represented an etiologically heterogeneous spectrum, comprising osteoarthritis-associated BME (Kellgren–Lawrence grade 1–3), BME related to subchondral insufficiency fracture without articular surface collapse, and primary bone marrow edema syndrome in patients without radiographic evidence of osteoarthritis. BME secondary to trauma, osteonecrosis, advanced structural collapse, or other identifiable secondary causes was excluded, as detailed above.

**Figure 1:**
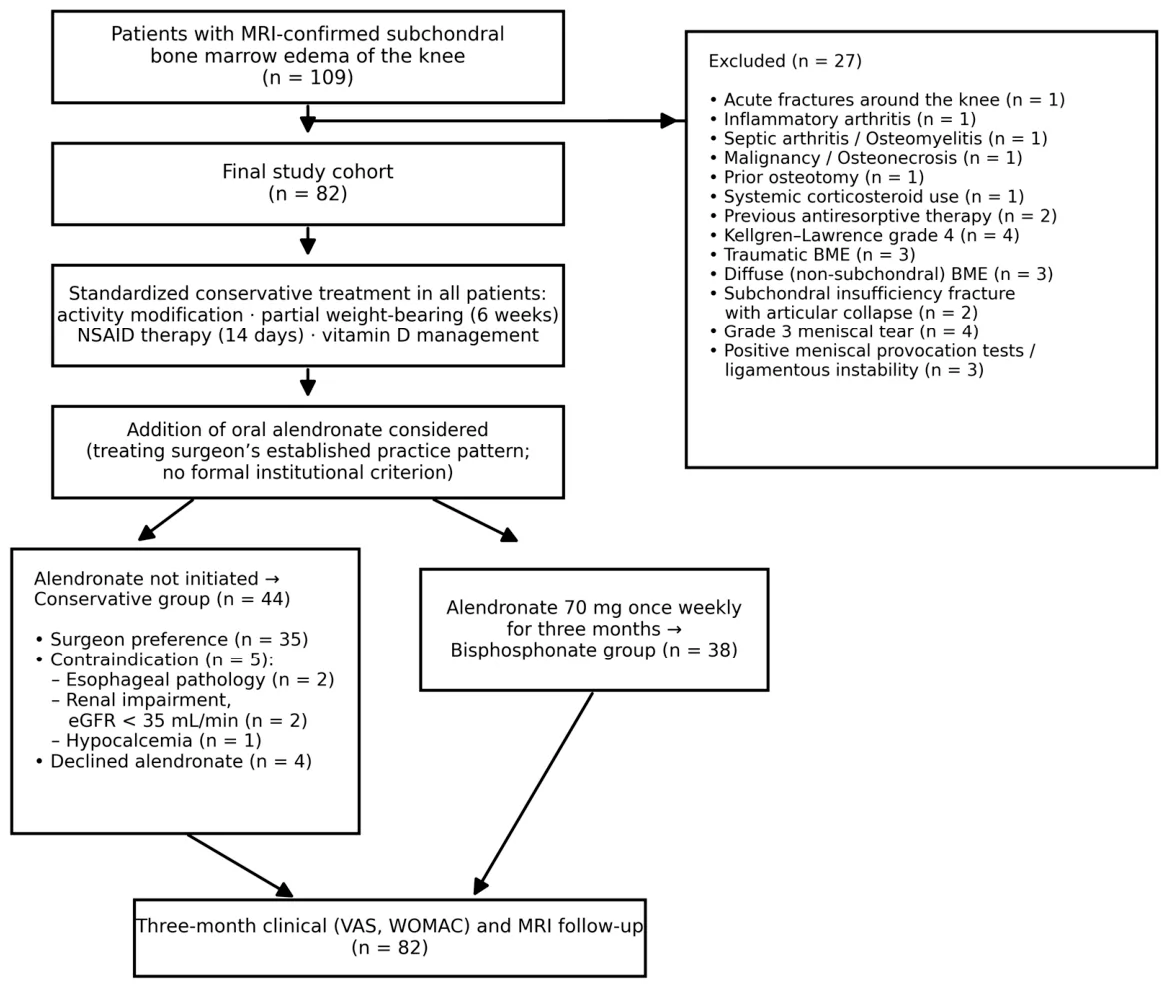
Flow diagram of patient selection and treatment allocation. MRI: magnetic resonance imaging; BME: bone marrow edema; NSAID: nonsteroidal anti-inflammatory drug; eGFR: estimated glomerular filtration rate; VAS: Visual Analog Scale; WOMAC: Western Ontario and McMaster Universities Osteoarthritis Index. Patients meeting more than one exclusion criterion were classified according to the first criterion met.

A total of 109 patients with MRI-confirmed subchondral bone marrow edema of the knee were retrospectively identified, of whom 27 were excluded after application of the eligibility criteria. The remaining 82 patients constituted the final study cohort (Figure 1).

Patients were categorized into two groups according to the treatment received. Oral bisphosphonate use was not uniformly adopted across treating surgeons in our institution; while some surgeons incorporated oral bisphosphonates into their management protocol, others preferred conservative treatment alone. Accordingly, 44 patients had been managed with standard conservative treatment (conservative group), whereas 38 patients had received conservative treatment combined with oral bisphosphonate therapy (bisphosphonate group).

### 2.3. Treatment Protocols

Patients in the conservative group were managed with activity modification, partial weight-bearing for six weeks, and NSAID therapy for 14 days. The choice of NSAID was based on the treating surgeon’s preference and patient characteristics. Patients in the bisphosphonate group had received the same conservative treatment protocol in addition to oral alendronate at a dose of 70 mg once weekly for three months. As vitamin D deficiency is frequently observed in patients with subchondral BME and may influence subchondral bone metabolism, serum 25-hydroxyvitamin D levels had been measured in all patients at baseline and managed uniformly across both groups to minimize its potential confounding effect on outcomes [8]. Patients with vitamin D deficiency (<20 ng/mL) or insufficiency (20–30 ng/mL) had received therapeutic supplementation, whereas those with sufficient levels (>30 ng/mL) had received maintenance-dose supplementation. This management approach represented the standardized institutional protocol for subchondral BME of the knee, and its core elements—the duration of partial weight-bearing, the 14-day course of NSAID therapy, the alendronate regimen, and the vitamin D management algorithm—remained unchanged throughout the study period (January 2020–January 2025). The only individualized element was the choice of NSAID agent, which was documented for all patients and formally compared between the groups. There was no formal institutional criterion mandating the addition of alendronate; the decision to add bisphosphonate therapy primarily reflected the established practice pattern of the treating surgeon rather than patient-specific severity indicators, with some surgeons routinely incorporating alendronate into their management of subchondral BME and others managing patients conservatively (n = 35 allocated to the conservative group on this basis). Irrespective of surgeon preference, alendronate was not initiated in patients with contraindications, namely esophageal pathology (n = 2), impaired renal function (estimated glomerular filtration rate < 35 mL/min; n = 2), or hypocalcemia (n = 1), and in 4 patients who declined bisphosphonate therapy. The resulting selection and allocation pathway is summarized in Figure 1. Clinical and radiological follow-up, including MRI examination, was performed three months after treatment initiation.

### 2.4. Clinical and Radiological Assessment

Clinical outcomes were assessed using the VAS for pain and the WOMAC [16,17]. The WOMAC has been validated for use in Turkish-speaking populations [18]. Baseline and three-month follow-up scores were obtained from patient records. The primary clinical outcome was the change in VAS score between baseline and the three-month follow-up. Secondary clinical outcomes included changes in WOMAC scores and the time required to return to full weight-bearing.

Radiological assessment was performed using baseline and three-month follow-up MRI examinations of the knee. All MRI examinations had been acquired on a 1.5-T scanner (Magnetom Aera; Siemens Healthineers, Erlangen, Germany). All MRI scans were independently reviewed by two musculoskeletal radiologists who were blinded to treatment allocation and clinical outcomes. In cases of disagreement, a consensus evaluation was performed. The maximal coronal and sagittal diameters of the bone marrow edema lesion were measured on fat-suppressed T2-weighted MRI images using a picture archiving and communication system workstation (IDS7, version 24.1; Sectra AB, Linköping, Sweden), and the edema area was estimated by multiplying these two diameters [19]. The percentage reduction in edema area between baseline and follow-up MRI examinations was calculated for each patient. Radiological response was categorized as complete regression (>75% reduction), partial regression (25–75% reduction), no change (<25% reduction), or progression (increase in edema area). The Kellgren–Lawrence grade of osteoarthritis and the presence of subchondral insufficiency fractures were also recorded [20]. 

### 2.5. Statistics

Statistical analyses were performed using IBM SPSS Statistics for Windows, Version 26.0 (IBM Corp., Armonk, NY, USA). Continuous variables were expressed as mean ± standard deviation (SD) or median (minimum–maximum) according to data distribution, whereas categorical variables were presented as frequencies and percentages. Normality of data distribution was assessed using the Shapiro–Wilk test. Comparisons between the two groups were performed using the independent-samples *t*-test for normally distributed continuous variables and the Mann–Whitney U test for non-normally distributed variables. Within-group comparisons between baseline and three-month follow-up measurements were performed using the paired-samples t-test or Wilcoxon signed-rank test, as appropriate. No a priori sample size calculation was performed, as this retrospective study included all eligible patients identified during the predefined five-year period. With the available group sizes (44 and 38 patients), the study had 80% power to detect a between-group difference of 0.63 standard deviations in the primary outcome (corresponding to approximately 0.85 VAS points) at a two-sided alpha of 0.05; smaller effects may therefore have gone undetected. Categorical variables were compared using the chi-square test or Fisher’s exact test, as appropriate. For contingency tables larger than 2 × 2 with sparse expected cell counts, the Fisher–Freeman–Halton exact test with Monte Carlo simulation was used. Inter-observer agreement for the categorical radiological response was assessed using Cohen’s kappa coefficient, and the intraclass correlation coefficient (ICC) was calculated for continuous BME area measurements. To identify factors associated with radiological response, a multivariate logistic regression analysis was performed. Radiological response was dichotomized as favorable (complete or partial regression) or unfavorable (no change or progression). Variables included in the model were treatment group and Kellgren–Lawrence grade, selected based on clinical relevance and the requirement for a minimum of 10 events per variable. Given the number of unfavorable radiological responses (n = 21), the events-per-variable criterion restricted the model to a maximum of two covariates. Kellgren–Lawrence grade was selected a priori as the covariate most likely to influence radiological response, reflecting the severity of underlying degenerative disease, whereas the remaining baseline characteristics (age, sex, BMI, BME location, baseline clinical scores, subchondral insufficiency fracture, and vitamin D status) were comparable between groups and could not be additionally included in the model. Results were expressed as odds ratios (OR) with 95% confidence intervals (CI). The primary outcome measure was the change in VAS score from baseline to three months, while secondary outcome measures included changes in WOMAC scores, time to return to full weight-bearing, and radiological response on follow-up MRI. A *p*-value of less than or equal to 0.05 (*p* ≤ 0.05) was considered statistically significant. The prespecified primary outcome was tested at this threshold without adjustment. To account for multiplicity among the six secondary between-group clinical comparisons, a Bonferroni-corrected significance threshold of *p* ≤ 0.008 (0.05/6) was additionally applied, and the secondary results are interpreted accordingly.

## 3. Results

### 3.1. Demographic and Baseline Characteristics

The final study cohort comprised 82 patients, of whom 44 were in the conservative group and 38 in the bisphosphonate group. The mean age of the entire cohort was 51.4 ± 8.7 years (range: 24–70 years), with a mean age of 52.1 ± 8.9 years in the conservative group and 50.6 ± 8.4 years in the bisphosphonate group (*p* = 0.41). No statistically significant differences were observed between the two groups with respect to sex, body mass index, affected side, or anatomical location of BME within the knee (all *p* > 0.05). Baseline VAS and WOMAC scores were comparable between groups (Table 1).

Vitamin D deficiency (<20 ng/mL) was present in 27 patients (61.4%) in the conservative group and 19 patients (50.0%) in the bisphosphonate group, while insufficiency (20–30 ng/mL) was identified in 10 (22.7%) and 9 (23.7%) patients, respectively. The proportion of patients with sufficient baseline vitamin D levels (>30 ng/mL) did not differ significantly between groups (*p* = 0.28). The Kellgren–Lawrence grade distribution, presence of subchondral insufficiency fractures, and anatomical location of BME did not differ significantly between groups at baseline (Table 1).

### 3.2. Nonsteroidal Anti-Inflammatory Drugs (NSAID) Utilization

Dexketoprofen trometamol was the most frequently prescribed NSAID in both groups, administered to 20 patients (45.5%) in the conservative group and 19 patients (50.0%) in the bisphosphonate group. Naproxen sodium was the second most commonly used agent in the conservative group (27.3%), whereas diclofenac sodium was more frequently preferred in the bisphosphonate group (23.7%). Despite these numerical differences, no statistically significant difference was detected in the distribution of individual NSAID agents or in the overall distribution between the two groups (*p* = 0.58), indicating comparable pharmacological management across both groups (Table 2).

### 3.3. Clinical Outcomes

Both groups demonstrated statistically significant reductions in VAS pain scores from baseline to the three-month follow-up (*p* < 0.001 for both). In the conservative group, the mean VAS score decreased from 7.3 ± 1.2 at baseline to 4.1 ± 1.4 at three months (mean reduction: 3.2 ± 1.3 points). In the bisphosphonate group, the mean VAS score decreased from 7.1 ± 1.3 at baseline to 3.1 ± 1.3 at three months (mean reduction: 4.0 ± 1.4 points). The between-group difference in VAS reduction was statistically significant in favor of the bisphosphonate group (*p* = 0.009) (Table 3).

Significant improvements in total WOMAC scores and all three subscales were observed in both groups at three months (*p* < 0.001 for both). In the conservative group, the total WOMAC score decreased from 58.4 ± 10.7 at baseline to 38.6 ± 9.8 at three months (mean reduction: 19.8 ± 8.6 points). In the bisphosphonate group, the total WOMAC score decreased from 57.9 ± 11.2 to 32.4 ± 9.1 (mean reduction: 25.5 ± 8.9 points). The between-group difference in total WOMAC score improvement was statistically significant in favor of the bisphosphonate group (*p* = 0.002) (Table 3).

Regarding the WOMAC subscales, the pain subscale score decreased from 12.8 ± 3.1 to 8.1 ± 2.7 in the conservative group, and from 12.5 ± 3.3 to 6.2 ± 2.4 in the bisphosphonate group, with a statistically significant between-group difference (*p* = 0.001). The stiffness subscale improved from 4.9 ± 1.6 to 3.2 ± 1.3 in the conservative group and from 4.7 ± 1.5 to 2.6 ± 1.2 in the bisphosphonate group (*p* = 0.03). The physical function subscale decreased from 40.7 ± 8.4 to 27.3 ± 7.2 in the conservative group and from 40.7 ± 8.6 to 23.6 ± 6.8 in the bisphosphonate group, with a statistically significant between-group difference (*p* = 0.02). Detailed WOMAC outcomes are presented in Table 3.

The median time to return to full weight-bearing was 9 weeks (range: 6–16 weeks) in the conservative group and 7 weeks (range: 6–13 weeks) in the bisphosphonate group (*p* = 0.021) (Table 3).

### 3.4. Radiological Outcomes

**Figure 2:**
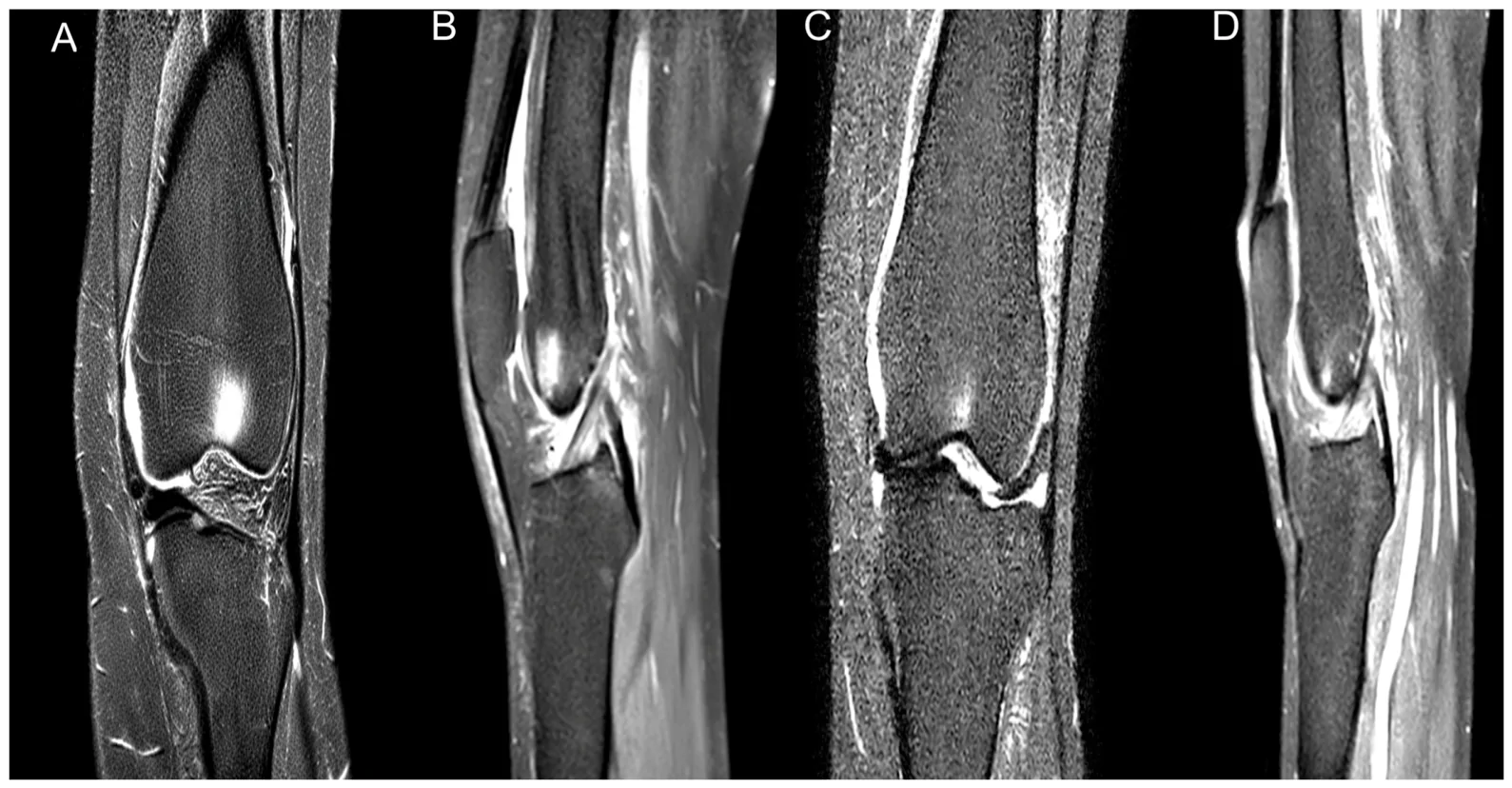
Representative MRI images of a 58-year-old patient in the bisphosphonate group demonstrating complete regression of subchondral bone marrow edema at three-month follow-up. (**A**) Baseline coronal fat-suppressed T2-weighted MRI showing extensive hyperintense signal changes in the medial femoral condyle consistent with subchondral BME. (**B**) Baseline sagittal fat-suppressed T2-weighted MRI demonstrating the craniocaudal extent of the edema. (**C**) Three-month follow-up coronal fat-suppressed T2-weighted MRI showing marked reduction (>75%) in BME area. (**D**) Three-month follow-up sagittal fat-suppressed T2-weighted MRI confirming near-complete resolution of the edema signal.

In the conservative group, complete regression of BME was observed in 8 patients (18.2%), partial regression in 20 patients (45.5%), no change in 12 patients (27.3%), and progression in 4 patients (9.1%). In the bisphosphonate group, complete regression was achieved in 16 patients (42.1%), partial regression in 17 patients (44.7%), no change in 4 patients (10.5%), and progression in 1 patient (2.6%). The overall distribution of radiological response categories differed significantly between the groups (Fisher–Freeman–Halton exact test with Monte Carlo simulation, *p* = 0.037) (Table 4). In a pairwise comparison, complete regression was more frequent in the bisphosphonate group (42.1% vs. 18.2%; Fisher’s exact test, *p* = 0.028). A representative case demonstrating complete regression of subchondral BME following oral bisphosphonate therapy is illustrated in Figure 2.

Inter-observer agreement between the two musculoskeletal radiologists was assessed for both categorical and continuous measurements. Agreement for the categorization of radiological response was excellent (κ = 0.84). The intraclass correlation coefficient for BME area measurements was also excellent (ICC = 0.91, 95% CI: 0.86–0.95).

### 3.5. Adverse Events

Overall adherence to oral alendronate therapy was high, with 34 of 38 patients (89.5%) completing at least 80% of the prescribed three-month treatment regimen. No serious adverse events related to bisphosphonate therapy were recorded during the three-month follow-up period. Mild upper gastrointestinal symptoms were reported by 4 patients (10.5%) in the bisphosphonate group, of whom 3 patients (7.9%) experienced nausea and 1 patient (2.6%) reported epigastric discomfort. All symptoms were mild in severity, transient in nature, and resolved spontaneously without requiring dose modification or treatment discontinuation. No cases of osteonecrosis of the jaw, atypical femoral fracture, hypocalcemia, or esophageal irritation were observed in either group.

### 3.6. Factors Associated with Radiological Response

For the purpose of regression analysis, radiological response was dichotomized as favorable (complete or partial regression) or unfavorable (no change or progression). In the multivariable model adjusted for Kellgren–Lawrence grade, bisphosphonate treatment was associated with higher odds of a favorable radiological response (OR: 3.66, 95% CI: 1.18–11.31, *p* = 0.024). Kellgren–Lawrence grade was not significantly associated with radiological response (OR: 0.65, 95% CI: 0.23–1.85, *p* = 0.417) (Table 5).

## 4. Discussion

The present study evaluated the clinical and radiological outcomes of conservative treatment with or without oral bisphosphonate therapy in patients with subchondral bone marrow edema of the knee. Our results demonstrated that the addition of oral alendronate to standard conservative treatment resulted in significantly greater reductions in pain and functional impairment, a shorter time to return to full weight-bearing, and superior radiological response compared to the conservative group. These findings support the hypothesis that oral bisphosphonate therapy provides meaningful clinical and radiological benefit in the management of subchondral BME of the knee, adding to the growing body of evidence suggesting a role for antiresorptive agents in this condition.

Subchondral BME is characterized by increased intraosseous pressure, impaired microcirculation, and elevated bone resorption activity, all of which contribute to the perpetuation of edema and pain [13,21]. By suppressing osteoclast activity, bisphosphonates are proposed to reduce subchondral stress, alleviate intraosseous hypertension, and promote a more favorable bone remodeling environment, thereby facilitating edema resolution, although the precise underlying mechanisms have not been fully elucidated [22,23,24]. Consistent with this proposed mechanism, bisphosphonates have also demonstrated efficacy in the treatment of osteonecrosis, where similar pathophysiological processes of impaired perfusion and elevated intraosseous pressure are implicated [25]. These mechanisms are consistent with the superior radiological response observed in our bisphosphonate group, where the rate of complete regression was more than twice that of the conservative group. The majority of previous studies have investigated intravenous bisphosphonate formulations, particularly zoledronic acid, ibandronate, and neridronate, which offer higher bioavailability and more predictable pharmacokinetics [9,10,11]. Our findings suggest that oral alendronate administered at a weekly dose of 70 mg for three months may provide comparable clinical and radiological benefits in the outpatient setting, with the added practical advantages of greater accessibility and lower cost compared to intravenous formulations.

The conservative and bisphosphonate groups were comparable at baseline with respect to age, sex, BMI, BME localization, KL grade, and baseline clinical scores, supporting the validity of between-group comparisons, and the demographic characteristics of the study cohort were broadly consistent with those reported in the existing literature on subchondral BME of the knee [11,12,13]. The distribution of NSAID agents was comparable between groups, with dexketoprofen trometamol being the most frequently prescribed agent in both, minimizing the potential for differential pharmacological confounding.

Both groups demonstrated clinically meaningful improvements in pain and functional outcomes at three months, with VAS reductions exceeding the accepted minimal clinically important difference of 2.0 points in both groups [26]. However, the bisphosphonate group achieved significantly greater reductions in VAS and total WOMAC scores, with superior improvement observed across all three WOMAC subscales, indicating that the benefit of oral alendronate extended beyond pain relief to encompass broader functional recovery. The significantly shorter time to return to full weight-bearing in the bisphosphonate group further supports the clinical relevance of these findings, as earlier mobilization is associated with reduced risk of deconditioning and faster return to daily activities.

Radiological findings corroborated the clinical results, with the bisphosphonate group demonstrating a significantly higher rate of complete BME regression at three months compared to the conservative group. These findings are broadly consistent with previous reports; Baier et al. [12] reported a complete regression rate of 40% with intravenous bisphosphonate therapy at three months, and the systematic review by Kon et al. [13] demonstrated that 36 of 78 bisphosphonate-treated patients achieved complete BME resolution within a mean of 3 months. The comparable complete regression rate observed in our bisphosphonate group suggests that oral alendronate may achieve similar radiological outcomes to intravenous formulations, with the added advantage of outpatient administration. Furthermore, in a multivariable model adjusted for Kellgren–Lawrence grade, bisphosphonate treatment remained associated with a favorable radiological response, while Kellgren–Lawrence grade itself was not, suggesting that this association may be independent of the degree of underlying osteoarthritis; nevertheless, given the observational treatment allocation, residual confounding cannot be excluded.

Vitamin D deficiency was prevalent in both groups at baseline, affecting approximately 60% of patients, which is consistent with previously reported rates in patients with subchondral BME [27]. Although robust evidence specifically addressing the efficacy of vitamin D supplementation in BME remains limited, emerging data suggest that optimizing vitamin D status may favorably influence subchondral bone metabolism and support the therapeutic response to antiresorptive agents [8,27]. In the present study, vitamin D management was applied uniformly across both groups according to baseline serum levels, thereby minimizing its potential confounding effect on the observed between-group differences and allowing the clinical and radiological outcomes to be more reliably attributed to bisphosphonate therapy.

The safety profile of oral alendronate was favorable, with mild and transient gastrointestinal symptoms observed in only 4 patients (10.5%), consistent with previously reported rates for short-term oral bisphosphonate use [28]. The absence of serious adverse events, including osteonecrosis of the jaw, atypical femoral fracture, or hypocalcemia, is reassuring and supports the tolerability of short-term oral alendronate therapy in this patient population.

Several methodological strengths enhance the validity of the present study. All MRI examinations were independently reviewed by two blinded musculoskeletal radiologists with excellent inter-observer agreement, and a standardized quantitative method was employed for BME area estimation, reducing the risk of subjective bias in radiological assessment. Furthermore, vitamin D status was systematically evaluated and managed uniformly across both groups, and the comparable distribution of NSAID agents minimized the potential for differential pharmacological confounding. From a clinical standpoint, the present findings indicate that oral alendronate at a weekly dose of 70 mg for three months, when added to standard conservative treatment, may offer a readily applicable and well-tolerated treatment strategy in the outpatient management of subchondral BME of the knee, without the logistical burden associated with intravenous bisphosphonate administration.

This study has several limitations. The retrospective, non-randomized, single-center design carries a risk of selection bias—treatment allocation reflected the treating surgeon’s preference rather than a standardized protocol—and may limit the generalizability of the findings. The cohort was etiologically heterogeneous, encompassing osteoarthritis-associated BME, insufficiency fracture–related BME, and primary bone marrow edema syndrome; although baseline distributions were comparable between groups, the small number of insufficiency fractures (n = 15) precluded subgroup analyses, and the findings therefore apply to non-traumatic subchondral BME as a whole rather than to any single underlying entity. Although the core conservative protocol remained unchanged throughout the study period, minor temporal variations in ancillary care cannot be excluded, and adherence to alendronate was ascertained from routine follow-up documentation rather than objective measures and may be overestimated. After Bonferroni correction of the secondary clinical comparisons, the differences in WOMAC stiffness, WOMAC physical function, and time to full weight-bearing retained only nominal significance and should be regarded as supportive findings. The small sample size restricted the multivariable model to two covariates and produced wide confidence intervals; residual confounding by variables that could not be adjusted for (including age, sex, BMI, baseline symptom severity, BME location, insufficiency fracture, vitamin D status, and symptom duration) cannot be excluded, propensity score–based methods were not feasible at this sample size, and the adjusted association between alendronate and radiological response should be regarded as exploratory. Finally, the three-month follow-up, although aligned with the treatment course, does not capture the long-term durability of the observed response.

Several future directions emerge from these findings. From a clinical perspective, weekly oral alendronate may be regarded as an accessible, low-cost, and well-tolerated adjunct to structured conservative treatment in patients with persistent symptomatic subchondral BME of the knee, provided that contraindications are respected; however, pending confirmation in randomized trials, it should complement rather than replace the core elements of conservative management. From a research perspective, adequately powered, multicenter, randomized placebo-controlled trials are needed to confirm the efficacy of oral alendronate, ideally incorporating standardized quantitative MRI-based lesion volumetry, head-to-head comparisons of oral and intravenous agents, and systematic evaluation of the optimal dose and treatment duration. Etiology-specific trials—separately addressing osteoarthritis-associated BME, insufficiency fracture–related BME, and primary bone marrow edema syndrome—together with extended follow-up assessing the durability of response, structural progression of osteoarthritis, and progression to arthroplasty, as well as formal cost-effectiveness analyses, would further clarify the role of oral bisphosphonates in joint-preserving treatment algorithms.

## 5. Conclusions

In conclusion, the addition of oral alendronate to standard conservative treatment resulted in significantly greater reductions in pain and functional impairment, a shorter time to return to full weight-bearing, and superior radiological response at three months compared to the conservative group in patients with subchondral bone marrow edema of the knee. Bisphosphonate treatment remained associated with a favorable radiological response after adjustment for Kellgren–Lawrence grade, while the safety profile of short-term oral alendronate was acceptable. These findings suggest that oral bisphosphonate therapy may represent a practical adjunct to conservative management in this patient population, although prospective randomized controlled trials with longer follow-up are needed to confirm these results and establish definitive treatment recommendations.

## Figures and Tables

**Table 1:** Baseline demographic and clinical characteristics of the study groups.

Variable	Conservative Group (n = 44)	Bisphosphonate Group (n = 38)	*p*
Age (years), mean ± SD	52.1 ± 8.9	50.6 ± 8.4	0.41
Female sex, n (%)	26 (59.1%)	21 (55.3%)	0.71
BMI (kg/m^2^), mean ± SD	27.3 ± 4.2	28.9 ± 3.7	0.07
Affected side (right), n (%)	27 (61.4%)	20 (52.6%)	0.41
BME location—medial femoral condyle	19 (43.2%)	14 (36.8%)	0.55
BME location—medial tibial plateau	15 (34.1%)	13 (34.2%)	0.99
BME location—lateral compartment	10 (22.7%)	11 (28.9%)	0.51
Baseline VAS score, mean ± SD	7.3 ± 1.2	7.1 ± 1.3	0.46
Baseline WOMAC total score, mean ± SD	58.4 ± 10.7	57.9 ± 11.2	0.83
Vitamin D deficiency, n (%)	27 (61.4%)	19 (50.0%)	0.28
KL grade 0–1, n (%)	19 (43.2%)	20 (52.6%)	0.38
KL grade 2–3, n (%)	25 (56.8%)	18 (47.4%)	0.38
Subchondral insufficiency fracture, n (%)	8 (18.2%)	7 (18.4%)	0.97

BMI: body mass index; BME: bone marrow edema; VAS: Visual Analog Scale; WOMAC: Western Ontario and McMaster Universities Osteoarthritis Index; KL: Kellgren–Lawrence. *p*-values were calculated using independent-samples *t*-test, Mann–Whitney U test, or chi-square test as appropriate.

**Table 2:** Distribution of nonsteroidal anti-inflammatory drugs (NSAID) agents used during treatment.

NSAID Agent	Conservative Group n (%)	Bisphosphonate Group n (%)	*p*
Dexketoprofen trometamol	20 (45.5%)	19 (50.0%)	0.67
Naproxen sodium	12 (27.3%)	7 (18.4%)	0.31
Diclofenac sodium	7 (15.9%)	9 (23.7%)	0.34
Celecoxib	5 (11.4%)	3 (7.9%)	0.57
Overall distribution			0.58

*p*-values for individual agents calculated using chi-square test or Fisher’s exact test as appropriate. Overall distribution compared using chi-square test. NSAID: nonsteroidal anti-inflammatory drugs.

**Table 3:** Clinical outcomes at baseline and three-month follow-up.

Outcome	Conservative Group (n = 44)	Bisphosphonate Group (n = 38)	*p*
WOMAC Total—Baseline	58.4 ± 10.7	57.9 ± 11.2	0.83
WOMAC Total—3 months	38.6 ± 9.8	32.4 ± 9.1	<0.001
WOMAC Total—Change	19.8 ± 8.6	25.5 ± 8.9	0.002
WOMAC Pain—Baseline	12.8 ± 3.1	12.5 ± 3.3	0.67
WOMAC Pain—3 months	8.1 ± 2.7	6.2 ± 2.4	0.001
WOMAC Stiffness—Baseline	4.9 ± 1.6	4.7 ± 1.5	0.54
WOMAC Stiffness—3 months	3.2 ± 1.3	2.6 ± 1.2	0.03
WOMAC Function—Baseline	40.7 ± 8.4	40.7 ± 8.6	0.99
WOMAC Function—3 months	27.3 ± 7.2	23.6 ± 6.8	0.02
Time to full weight-bearing weeks (median)	9 (6–16)	7 (6–13)	0.021

Between-group comparisons were performed using an independent-samples *t*-test or Mann–Whitney U test. After Bonferroni correction for the six secondary between-group comparisons at follow-up (adjusted significance threshold *p* ≤ 0.008), the differences in WOMAC total score at three months, WOMAC total score change, and WOMAC pain at three months remained statistically significant, whereas the differences in WOMAC stiffness, WOMAC physical function, and time to full weight-bearing were nominally significant only.

**Table 4:** Radiological response on follow-up MRI at three months.

Radiological Response	Conservative n (%)	Bisphosphonate n (%)
Complete regression	8 (18.2%)	16 (42.1%)
Partial regression	20 (45.5%)	17 (44.7%)
No change	12 (27.3%)	4 (10.5%)
Progression	4 (9.1%)	1 (2.6%)

Overall radiological response distribution was compared using the Fisher–Freeman–Halton exact test with Monte Carlo simulation; *p* = 0.037.

**Table 5:** Multivariate logistic regression analysis for factors associated with favorable radiological response.

Variable	OR	95% CI	*p*
Treatment group (bisphosphonate vs. conservative)	3.66	1.18–11.31	0.024
KL grade (2–3 vs. 0–1)	0.65	0.23–1.85	0.417

OR: odds ratio; CI: confidence interval; KL: Kellgren–Lawrence. Favorable radiological response defined as complete or partial regression on follow-up MRI.

## Data Availability

The data can be obtained from the corresponding author upon request.

## Supplementary Materials

The following supporting information can be downloaded at: https://www.mdpi.com/article/10.3390/jcm15176529/s1. Table S1: STROBE checklist for cohort studies.

## Abbreviations

The following abbreviations are used in this manuscript:

BME	Bone marrow edema
BMI	Body mass index
CI	Confidence interval
ICC	Intraclass correlation coefficient
KL	Kellgren–Lawrence
MRI	Magnetic resonance imaging
NSAID	Nonsteroidal anti-inflammatory drug
OR	Odds ratio
SD	Standard deviation
VAS	Visual analog scale
WOMAC	Western Ontario and McMaster Universities Osteoarthritis Index

## References

[1] Villari E., Digennaro V., Panciera A., Ferri R., Benvenuti L., Faldini C. (2024). Bone marrow edema of the knee: A narrative review. Arch. Orthop. Trauma Surg..

[2] Malghem J., Lecouvet F., Vande Berg B., Kirchgesner T., Omoumi P. (2023). Subchondral insufficiency fractures, subchondral insufficiency fractures with osteonecrosis, and other apparently spontaneous subchondral bone lesions of the knee—Pathogenesis and diagnosis at imaging. Insights Imaging.

[3] Zanetti M., Bruder E., Romero J., Hodler J. (2000). Bone marrow edema pattern in osteoarthritic knees: Correlation between MR imaging and histologic findings. Radiology.

[4] Bonadio M.B., Filho A.G.O., Helito C.P., Stump X.M., Demange M.K. (2017). Bone marrow lesion: Image, clinical presentation, and treatment. Magn. Reson. Insights.

[5] Ip S., Sayre E.C., Guermazi A., Nicolaou S., Wong H., Thorne A., Singer J., Kopec J.A., Esdaile J.M., Cibere J. (2011). Frequency of bone marrow lesions and association with pain severity: Results from a population-based symptomatic knee cohort. J. Rheumatol..

[6] Guermazi A., Niu J., Hayashi D., Roemer F.W., Englund M., Neogi T., Aliabadi P., McLennan C.E., Felson D.T. (2012). Prevalence of abnormalities in knees detected by MRI in adults without knee osteoarthritis: Population based observational study (Framingham Osteoarthritis Study). BMJ.

[7] Felson D.T., McLaughlin S., Goggins J., LaValley M.P., Gale M.E., Totterman S., Li W., Hill C., Gale D. (2003). Bone marrow edema and its relation to progression of knee osteoarthritis. Ann. Intern. Med..

[8] Horas K., Fraissler L., Maier G., Jakob F., Seefried L., Konrads C., Rudert M., Walcher M. (2017). High prevalence of vitamin D deficiency in patients with bone marrow edema syndrome of the foot and ankle. Foot Ankle Int..

[9] Lurati A., Laria A., Faggioli P., Tamburello A., Castelnovo L., Mazzone A. (2020). Neridronate in bone marrow edema syndrome. Efficacy and safety of two therapeutic regimens. Beyond Rheumatol..

[10] Seefried L., Genest F., Baumann J., Heidemeier A., Meffert R., Jakob F. (2022). Efficacy of zoledronic acid in the treatment of nonmalignant painful bone marrow lesions: A triple-blind, randomized, placebo-controlled phase III clinical trial (ZoMARS). J. Bone Miner. Res..

[11] Küchler L., Fehr T., Jeker R. (2020). Pain control with ibandronate for bone marrow oedema of the knee. Swiss Med. Wkly..

[12] Baier C., Schaumburger J., Götz J., Heers G., Schmidt T., Grifka J., Beckmann J. (2013). Bisphosphonates or prostacyclin in the treatment of bone-marrow oedema syndrome of the knee and foot. Rheumatol. Int..

[13] Kon E., Ronga M., Filardo G., Farr J., Madry H., Milano G., Andriolo L., Shabshin N. (2016). Bone marrow lesions and subchondral bone pathology of the knee. Knee Surg. Sports Traumatol. Arthrosc..

[14] Paraskevopoulos K., Keskinis A., Vasios I.S., Makiev K.G., Tilkeridis K., Drosos G.I., Ververidis A.N. (2023). Comparison of various treatment modalities for the management of bone marrow edema syndrome/transient osteoporosis in men and non-pregnant women: A systematic review. Osteoporos. Int..

[15] Li S., Yu H., Long S., Li J., He Y., Zheng X., Yang S., Tang Y., Xie Q., Zheng W. (2023). Research advances in the treatment of bone marrow edema syndrome. J. Clin. Densitom..

[16] Huskisson E.C. (1974). Measurement of pain. Lancet.

[17] Bellamy N., Buchanan W.W., Goldsmith C.H., Campbell J., Stitt L.W. (1988). Validation study of WOMAC: A health status instrument for measuring clinically important patient relevant outcomes to antirheumatic drug therapy in patients with osteoarthritis of the hip or knee. J. Rheumatol..

[18] Tüzün E.H., Eker L., Aytar A., Daşkapan A., Bayramoğlu M. (2005). Acceptability, reliability, validity and responsiveness of the Turkish version of WOMAC osteoarthritis index. Osteoarthr. Cartil..

[19] Koenig F.R.M., Raudner M., Juras V., Szomolanyi P., Vetchy V., Kittinger J., Zadeh E.S., Watzenböck M.L., Trattnig S. (2025). Bone marrow edema-like signal after cartilage repair does not affect outcomes in a five-year follow-up. Eur. Radiol..

[20] Kellgren J.H., Lawrence J.S. (1957). Radiological assessment of osteo-arthrosis. Ann. Rheum. Dis..

[21] Manara M., Varenna M. (2014). A clinical overview of bone marrow edema. Reumatismo.

[22] Littman J., Gil H., Aaron R. (2023). Spontaneous bone marrow edema: Perfusion abnormalities and treatment with surgical decompression. Int. J. Mol. Sci..

[23] Jensen P.R., Andersen T.L., Chavassieux P., Roux J.P., Delaisse J.M. (2021). Bisphosphonates impair the onset of bone formation at remodeling sites. Bone.

[24] Deveza L.A., Bierma-Zeinstra S.M.A., van Spil W.E., Oo W.M., Saragiotto B.T., Neogi T., van Middelkoop M., Hunter D.J. (2018). Efficacy of bisphosphonates in specific knee osteoarthritis subpopulations: Protocol for an OA Trial Bank systematic review and individual patient data meta-analysis. BMJ Open.

[25] Ramachandran M., Ward K., Brown R.R., Munns C.F., Cowell C.T., Little D.G. (2007). Intravenous bisphosphonate therapy for traumatic osteonecrosis of the femoral head in adolescents. J. Bone Jt. Surg. Am..

[26] Farrar J.T., Young J.P., LaMoreaux L., Werth J.L., Poole M.R. (2001). Clinical importance of changes in chronic pain intensity measured on an 11-point numerical pain rating scale. Pain.

[27] Oehler N., Mussawy H., Schmidt T., Rolvien T., Barvencik F. (2018). Identification of vitamin D and other bone metabolism parameters as risk factors for primary bone marrow oedema syndrome. BMC Musculoskelet. Disord..

[28] Zhou M., Zheng Y., Li J., Wu J., Xu W., Cui L., Yao W., Liu Y. (2016). Upper gastrointestinal safety and tolerability of oral alendronate: A meta-analysis. Exp. Ther. Med..

