# Supplementary material for: Clinical and Radiological Outcomes of Oral Bisphosphonate Therapy Combined with Conservative Treatment in Subchondral Bone Marrow Edema of the Knee: A Retrospective Cohort Study
Source: J Clin Med. 2026 Aug 24;15(17):6529. doi: 10.3390/jcm15176529 (PMC13566092; doi:10.3390/jcm15176529)
Supplement: Supplementary file 1 [file jcm-15-06529-s001.zip › jcm-4505890-supplementary.pdf]

Table S1. STROBE checklist for cohort studies.

Manuscript ID: jcm-4505890.

Item No	Item	Recommendation	Page No
1 (a)	**Title and abstract**	Indicate the study's design with a commonly used term in the title or the abstract	1 (title: "A Retrospective Cohort Study"; abstract: "This retrospective cohort study")
1 (b)		Provide in the abstract an informative and balanced summary of what was done and what was found	1
2	**Background/rationale**	Explain the scientific background and rationale for the investigation being reported	1–2 (Introduction)
3	**Objectives**	State specific objectives, including any prespecified hypotheses	2 (final paragraph of Introduction: aim, hypothesis, primary and secondary outcomes)
4	**Study design**	Present key elements of study design early in the paper	3 (Study Design)
5	**Setting**	Describe the setting, locations, and relevant dates, including periods of recruitment, exposure, follow-up, and data collection	3, 5 (single tertiary center; January 2020–January 2025; data extraction after ethics approval of 2 July 2025; three-month follow-up)
6 (a)	**Participants**	Give the eligibility criteria, and the sources and methods of selection of participants; describe methods of follow-up	3–4 (Patient Selection: inclusion/exclusion criteria, electronic medical record identification; follow-up at three months)
6 (b)		For matched studies, give matching criteria and number of exposed and unexposed	Not applicable (unmatched cohort)
7	**Variables**	Clearly define all outcomes, exposures, predictors, potential confounders, and effect modifiers; give diagnostic criteria, if applicable	5–6 (Treatment Protocols; Clinical and Radiological Assessment; Statistics: primary and secondary

Item No	Item	Recommendation	Page No
			outcomes, covariates)
8	**Data sources/measurement**	For each variable of interest, give sources of data and details of methods of assessment (measurement); describe comparability of assessment methods if there is more than one group	5 (VAS and WOMAC instruments; standardized MRI protocol; BME area measurement; identical assessment in both groups)
9	**Bias**	Describe any efforts to address potential sources of bias	3–5 (exclusion of secondary causes and bone-acting medication; standardized protocol; uniform vitamin D management; two blinded radiologists with consensus adjudication); 12 (Limitations)
10	**Study size**	Explain how the study size was arrived at	6 (Statistics: inclusion of all eligible patients in the predefined period; 80% power to detect d = 0.63)
11	**Quantitative variables**	Explain how quantitative variables were handled in the analyses; if applicable, describe which groupings were chosen and why	5–6 (Kellgren–Lawrence grouping 0–1 vs 2–3; radiological response categories; dichotomization of response for regression)
12 (a)	**Statistical methods**	Describe all statistical methods, including those used to control for confounding	5–6 (Statistics: t-test/Mann–Whitney; chi-square/Fisher; Fisher–Freeman–Halton with Monte Carlo; Bonferroni for secondary comparisons; multivariable logistic regression adjusted for

Item No	Item	Recommendation	Page No
			Kellgren–Lawrence grade)
12 (b)		Describe any methods used to examine subgroups and interactions	6; 12 (subgroup analyses precluded by small number of insufficiency fractures, stated in Limitations)
12 (c)		Explain how missing data were addressed	3 (complete baseline and follow-up data were an eligibility requirement; patients without three-month MRI were excluded)
12 (d)		If applicable, explain how loss to follow-up was addressed	3 (analysis restricted to patients with complete three-month follow-up)
12 (e)		Describe any sensitivity analyses	6 (Bonferroni-corrected interpretation of secondary comparisons)
13 (a)	**Participants**	Report numbers of individuals at each stage of study — e.g., numbers potentially eligible, examined for eligibility, confirmed eligible, included in the study, completing follow-up, and analysed	4 (109 identified, 27 excluded, 82 analysed; Figure 1)
13 (b)		Give reasons for non-participation at each stage	4 (Figure 1: itemized exclusion reasons; reasons for non-initiation of alendronate)
13 (c)		Consider use of a flow diagram	4 (Figure 1)
14 (a)	**Descriptive data**	Give characteristics of study participants (e.g., demographic, clinical, social) and information on exposures and potential confounders	7 (Table 1; Table 2)
14 (b)		Indicate number of participants with missing data for each variable of interest	3 (no missing data by design; complete-case cohort)
14 (c)		Summarise follow-up time (e.g., average and total amount)	5 (uniform three-month follow-up in all patients)

Item No	Item	Recommendation	Page No
15	**Outcome data**	Report numbers of outcome events or summary measures over time	8–9 (Tables 3 and 4; Results: clinical and radiological outcomes)
16 (a)	**Main results**	Give unadjusted estimates and, if applicable, confounder-adjusted estimates and their precision (e.g., 95% confidence interval); make clear which confounders were adjusted for and why they were included	8–10 (between-group comparisons; adjusted OR 3.66, 95% CI 1.18–11.31 for treatment, adjusted for Kellgren–Lawrence grade; rationale on p. 6)
16 (b)		Report category boundaries when continuous variables were categorized	5–6 (Kellgren–Lawrence 0–1 vs 2–3; vitamin D <20 / 20–30 / >30 ng/mL)
16 (c)		If relevant, consider translating estimates of relative risk into absolute risk for a meaningful time period	9 (absolute proportions of radiological response categories at three months, Table 4)
17	**Other analyses**	Report other analyses done — e.g., analyses of subgroups and interactions, and sensitivity analyses	8–10 (Bonferroni-corrected interpretation, Table 3 footnote; pairwise comparison of complete regression; inter-observer agreement)
18	**Key results**	Summarise key results with reference to study objectives	10 (first paragraph of Discussion)
19	**Limitations**	Discuss limitations of the study, taking into account sources of potential bias or imprecision; discuss both direction and magnitude of any potential bias	12 (Limitations paragraph)
20	**Interpretation**	Give a cautious overall interpretation of results considering objectives, limitations, multiplicity of analyses, results from similar studies, and other relevant evidence	10–13 (Discussion, including future directions paragraph and Conclusions)
21	**Generalisability**	Discuss the generalisability (external validity) of the study results	12 (Limitations: single-center design; etiologically

Item No	Item	Recommendation	Page No
			heterogeneous non-traumatic BME population)
22	**Funding**	Give the source of funding and the role of the funders for the present study and, if applicable, for the original study on which the present article is based	13 (Funding statement; Acknowledgments)

Note: An Explanation and Elaboration article discusses each checklist item and gives methodological background and published examples of transparent reporting. The STROBE checklist is best used in conjunction with this article (freely available on the websites of PLoS Medicine, Annals of Internal Medicine, and Epidemiology). Information on the STROBE Initiative is available at www.strobe-statement.org.
